# Evaluating the implementation of a national clinical programme for diabetes to standardise and improve services: a realist evaluation protocol

**DOI:** 10.1186/s13012-016-0464-9

**Published:** 2016-07-28

**Authors:** S. McHugh, M. L. Tracey, F. Riordan, K O’Neill, N. Mays, P. M. Kearney

**Affiliations:** 1 Department of Epidemiology & Public Health, Western Gateway Complex, University College Cork, Western Rd, Cork, Ireland; 2 Department of Health Services Research & Policy, London School of Hygiene and Tropical Medicine, 15-17 Tavistock Place, London, WC1H 9SH UK

**Keywords:** Realist evaluation, Protocol, Diabetes, Implementation

## Abstract

**Background:**

Over the last three decades in response to the growing burden of diabetes, countries worldwide have developed national and regional multifaceted programmes to improve the monitoring and management of diabetes and to enhance the coordination of care within and across settings. In Ireland in 2010, against a backdrop of limited dedicated strategic planning and engrained variation in the type and level of diabetes care, a national programme was established to standardise and improve care for people with diabetes in Ireland, known as the National Diabetes Programme (NDP). The NDP comprises a range of organisational and service delivery changes to support evidence-based practices and policies. This realist evaluation protocol sets out the approach that will be used to identify and explain which aspects of the programme are working, for whom and in what circumstances to produce the outcomes intended.

**Methods/design:**

This mixed method realist evaluation will develop theories about the relationship between the context, mechanisms and outcomes of the diabetes programme. In stage 1, to identify the official programme theories, documentary analysis and qualitative interviews were conducted with national stakeholders involved in the design, development and management of the programme. In stage 2, as part of a multiple case study design with one case per administrative region in the health system, qualitative interviews are being conducted with frontline staff and service users to explore their responses to, and reasoning about, the programme’s resources (mechanisms). Finally, administrative data will be used to examine intermediate implementation outcomes such as service uptake, acceptability, and fidelity to models of care.

**Discussion:**

This evaluation is using the principles of realist evaluation to examine the implementation of a national programme to standardise and improve services for people with diabetes in Ireland. The concurrence of implementation and evaluation has enabled us to produce formative feedback for the NDP while also supporting the refinement and revision of initial theories about how the programme is being implemented in the dynamic and unstable context of the Irish healthcare system.

**Electronic supplementary material:**

The online version of this article (doi:10.1186/s13012-016-0464-9) contains supplementary material, which is available to authorized users.

## Background

Diabetes is a major public health and health service challenge worldwide with global prevalence estimated to increase from 2.8 % in 2000 to 4.4 % in 2030, an increase from 171 million people to 366 million people in 30 years [1]. The most recent Global Burden of Disease study estimates that diabetes is the seventh leading cause of years lived with disability worldwide [2]. Diabetes is associated with reduced quality of life and life expectancy [3, 4]. There are also significant societal and health service costs associated with the condition; global health expenditure on diabetes was estimates to be at least US$376 billion in 2010, rising to US$490 billion by 2030 [5].

### Optimal diabetes care

The need for organised coordinated implementation of strategies to improve diabetes care and reduce disease burden has long been recognised. In 1989, health departments from across Europe, including Ireland, signed the St. Vincent Declaration, a set of standards and goals to improve diabetes care [6]. The onus was placed on individual governments to implement strategies to meet the agreed targets. Over the next three decades, a number of countries developed national and regional multidimensional programmes to improve the monitoring and management of diabetes and to enhance the coordination of care within and across settings [7–10].

Consensus exists on what constitutes good quality diabetes care. Substantial evidence supports treatments to manage diabetes and slow the progression of complications [11–15]. National and international guidelines recommend the regular monitoring and management of blood glucose levels, blood pressure, kidney function, body mass index and smoking status, as well as routine foot surveillance, retinopathy screening and patient self-management education [16–18]. At a system level, the organisational features of high-quality diabetes care include regular review, patient registration and recall [19–21]. There has been a shift towards multidisciplinary shared management of complex patients across primary and secondary care settings, and structured management of stable diabetes in primary care with suitable organisational support [21, 22]. While quality improvement strategies targeting professionals and patients improve diabetes care and patient outcomes, strategies which target the entire system of chronic disease management, such as case management, team changes and patient registry, are associated with the largest benefits [23].

### Diabetes services in Ireland

Over the last two decades in Ireland, a plethora of policies and reports have repeatedly called for evidence-based service developments seen in other countries [24]. A number of diabetes initiatives have emerged, led by healthcare professionals, to improve diabetes care at a local level but with inconsistent implementation of a comprehensive diabetes service nationally. The balance of care between primary and secondary care settings varies and includes traditional hospital-based management, shared care between GPs and hospitals, and structured primary care-led management. Care in general practice ranges from ad hoc opportunistic management to structured care characterised by patient registration, regular recall and review coordinated by practice nurses [25]. A national survey of GPs reported that less than half used a patient register and diabetes guidelines or engaged in routine recall of patients with diabetes. Less than 10 % had a formal shared protocol or ever had a joint meeting with the hospital diabetes team. There was deficient access to allied health services such as podiatry, dietetics and eye screening [26]. Within the hospitals, not all diabetes services are led by an endocrinologist. Endocrinology-led services in Ireland had more developed subspecialty clinics and greater access to specialist allied health professionals. However, waiting times for these services were longer and discharge rates to primary care were lower than for non-specialty led services [27]. The provision of structured diabetes care in general practice and shared care between settings has produced favourable results in Ireland in terms of processes and outcomes of care [28–31]. However, these models of care are not common-place and there is a dearth of information on the quality of routine diabetes management at a national level.

### The National Diabetes Programme: a complex intervention to standardise care

Against this backdrop of variation in the type and quality of diabetes care, and a lack of dedicated strategic national planning and programme implementation, in 2010, a clinical programme for diabetes was established to standardise and improve care for people with diabetes in Ireland, known as the National Diabetes Programme (NDP) [32]. The NDP was one of a number of clinical care programmes set up under the auspices of the Directorate of Clinical Strategy and Programme in the Health Service Executive (HSE), the national health service in Ireland. The overarching goals of these programmes are to improve access to services, quality and safety of care, and cost effectiveness. These goals are achieved by bringing together representatives from various clinical disciplines to develop standardised patient pathways and evidence-based models of care [32]. Similar to the other programmes, the diabetes programme has a defined governance structure with a national clinical lead and programme manager, a clinical advisory group, and a national working group with the joint involvement of healthcare providers in primary, secondary and tertiary care [33]. There are also four regional Diabetes Services Implementation Groups (DSIGs), which are multidisciplinary regional networks established to inform the development and implementation of the National Diabetes Programme.

**Fig. 1:**
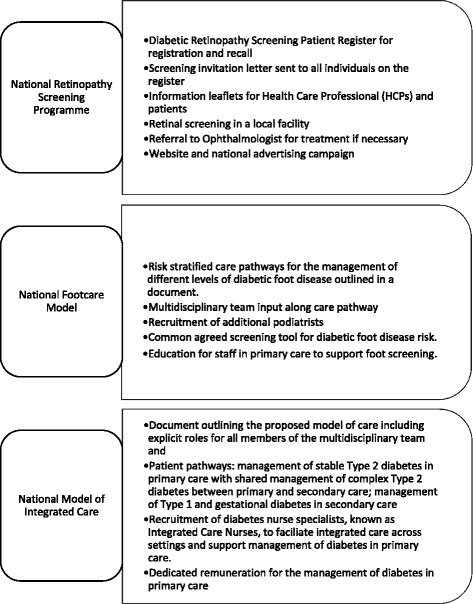
Intervention components of the National Programme for Diabetes

The specific aim of the NDP is to ‘save the eyes, limbs and lives of people with diabetes’ [33]. Like other large-scale service delivery innovations [34], a change in patient outcomes was to be achieved through the coordinated reorganisation of existing services, and the introduction of new services and supports for people with diabetes. Dedicated work streams were established for the implementation of a national retinopathy screening programme, a national model of care for the screening and treatment of diabetic foot disease, and a national model of integrated care for the management of diabetes across primary, secondary and tertiary care settings (Fig. 1).

Service innovations such as the NDP lead to both intended and unintended consequences. Evaluation provides an opportunity not only to identify and track these but also to identify multilevel barriers to implementation and conditions that make success and sustainability more likely. However, process evaluation is difficult to apply to complex services spanning organisational boundaries [35]. The NDP represents a number of complex interventions introduced into a complex social system, making it an appropriate subject for a realist evaluation. The realist evaluation approach, developed by Pawson and Tilley, goes beyond looking at whether a programme works or not, to try and understand which aspects of the programme work, for whom, in what circumstances [36, 37]. There is an inherent acknowledgement that a programme will work differently in different contexts; the aim is to understand what it is about a programme that leads to different outcomes [38].

According to Pawson and colleagues, complex service interventions are based on an underlying hypothesis of how the intervention will bring about an outcome [37, 39]. The first step of a realist evaluation is to identify and articulate these theories, known as programme theories. A programme provides a resource, an opportunity or a constraint, that influences the decision-making process of its intended target group. It is this decision-making process that determines whether an outcome is achieved or not; complex interventions are active, that is they only work through stakeholders’ reasoning and responses. This underlying interaction between what a programme provides and the reasoning of its intended targets is known as a mechanism. Understanding and explaining the often invisible implicit mechanisms are core functions of a realist evaluation [37, 40]. Mechanisms are argued to be triggered, to a greater or lesser extent in certain favourable and unfavourable contexts, leading to intended and unintended outcomes. The programme theory articulates a theoretical relationship between a context, mechanism and outcome, known as a ‘C-M-O’ configuration [37].

In this paper, we present the protocol for an evaluation of the NDP that adopts a realist approach. The aim of the evaluation is to identify and explain which aspects of the programme are working (or not working), for whom and in what circumstances to produce outcomes. The evaluation will examine three ongoing work streams of the NDP which have been prioritised since its inception in 2010: the introduction of a national diabetic retinopathy screening programme (initiated in 2013); the establishment of a national model of foot care for people with diabetes (staff recruited in 2013); and the development of a national model of integrated care for diabetes (staff recruited in 2013). The aim of this paper is to outline in detail the stages, methods and data being used in the evaluation, as well as some of the challenges to, and opportunities for, providing formative feedback to the NDP.

## Methods/design

**Fig. 2:**
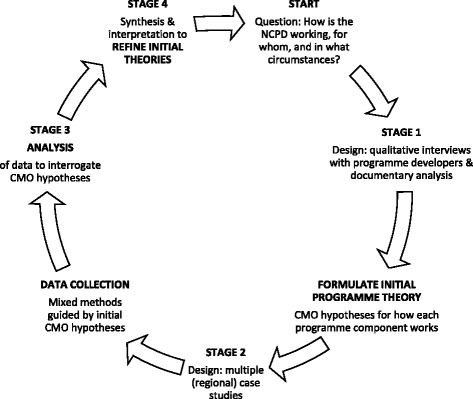
Realist research cycle (adapted with permission from [63])

This prospective evaluation follows the research stages outlined by Pawson and Tilley: (1) elicit and formulate the programme theory underlying the NDP and its work streams (national retinopathy screening programme, national foot care model and national model of integrated care), (2) collect data to test these initial theories, (3) analyse data to interrogate the theories and (4) interpret analysis to refine or revise the initial programme theories [36] (Fig. 2).

Realist evaluation is method neutral and most studies employ both quantitative and qualitative research methods [41–44]. We are using mixed methods depending on the stage of the study and the theory component (context, mechanism or outcome) under scrutiny; for example documentary analysis and qualitative interviews are considered useful for identifying the official programme theory and context, qualitative interviews are also appropriate for exploring stakeholders’ responses to and reasoning about the programme (mechanisms), and quantitative administrative data allow examination of outcomes such as service uptake [37]. Table 1 summarises the methods being used during data collection at stage 1 and stage 2.

**Table 1:** Data collection during stages 1 and 2 to formulate and refine programme theories

Stage	Methods
Stage 1: Elicit and formulate the programme theory	• Documentary analysis of published and unpublished documents pertaining to the establishment, development and implementation of the National Programme for Diabetes and the three programme interventions. • Semi-structured interviews with national level programme developers (*n* = 19).
Stage 2: Data Collection	Implementation of the National Programme for Diabetes • Multiple case study design (*n* = 4 cases) • Semi-structured interviews with theoretically sampled stakeholders in each area (*n* = approx. 15 per case).
	Further data for each case will be gathered through a number of sub-studies conducted at a local and national level to build a rich case description and allow for embedded analysis of each programme component.
	Retinopathy screening programme • Audit of registration, consent and uptake among a convenience sample of two large primary care centres (*n* = 22 GPs, approx. 600 people with diabetes) and a smaller rural practice in one region (*n* = 2 GPs, approx. 100 people with diabetes) • Semi-structured interviews with people with diabetes from those practices
	National foot care model • Semi-structured interviews with podiatrists including those recruited as part of the programme. • Cross-sectional study of administrative activity data submitted by podiatrists as part of the National Programme for Diabetes.
	National model of integrated care • National survey of Diabetes Nurse Specialists (DNSs) including those recruited as part of the programme. • Follow-up interviews and focus groups • Cross-sectional analysis of administrative activity data submitted by DNSs recruited as part of the National Programme for Diabetes

### Stage 1: elicit and formulate the programme theory

#### Design

As a theory-based evaluation approach, the first step of the realist evaluation is to clarify the ‘programme theory’; that is how the NDP and its three work streams are expected to cause or contribute to outcomes. The programme theory articulates the relationship between a context, mechanism and outcomes of the national programme components, known as ‘C-M-O’ configurations.

#### Data collection

Three data sources were used to develop the initial programme theory. Firstly, a documentary analysis was carried out to establish the official programme theory, expectations and rationale for establishing the Programme. Documents included published and unpublished material such as strategy documents from interest groups, media coverage, press releases, national service plans, NDP website, and official documentation on the role and function of the programmes. An additional file outlines the type and source of documents (see Additional file 1). These data were also useful for mapping the context in which the programme was being designed and implemented.

Secondly and concurrently, we conducted qualitative interviews with a purposive sample of stakeholders involved at a national level in the design, development and management of the Programme. All members of the national diabetes working group were invited to take part (membership between July 2014 and January 2015) as well as former clinical leads and programme managers. The national diabetes working group comprises representatives from endocrinology, general practice, diabetes nurse specialists and practice nurses, dietetics, podiatry, community pharmacy, public health, patient advocacy and health service management. Members also represent different parts of the country. Following an initial invitation via email, all participants were contacted individually by a member of the research team (MT) to outline the study and arrange a convenient time and place for interview.

A semi-structured topic guide was developed informed by initial findings from the documentary analysis and previously published realist evaluations [38, 42]. The topic guide was piloted with a convenience sample of two participants involved in diabetes care, who were not members of the national working group but were aware of the work of the Programme. Minor amendments were made to the prompts and probes used within the topic guide. The topic guide addressed participants’ role in the Programme, why the programme was established, the planned changes and how they were being implemented, progress to date, anticipated barriers and facilitators, and expected outcomes (see Additional file 2).

Face-to-face interviews were conducted (by MT) with 19 participants between July 2014 and January, 2015 (average duration 1 h). Participants received an information sheet and signed a consent form prior to the interview. These participants are implementers within their own local diabetes service as well as being involved in the design of programme at a national level. Thus, in addition to discussing planned implementation and expected outcomes (official programme theory), participants discussed their own experience of implementation, perceived outcomes in their area, and barriers encountered. Data collection and analysis were iterative to allow the gathering of further data on emergent themes and the topic guide was modified to accommodate emergent lines of inquiry.

Thirdly, following a presentation of preliminary findings, a short survey was conducted among attendees at the annual conference held by the NDP (November, 2015). Attendees, including healthcare professionals, patient representatives, health service managers and policy makers involved in or affected by the national programme, were invited to complete open-ended questions about which aspects of the national programme were working well, which aspects were not working as well, and why. Respondents were asked to indicate their professional role and the area of the country in which they worked. Thirty attendees completed the questionnaire (approximately 25 % response rate). Gathering the opinions of those involved in implementation from around the country allowed for further refinement and corroboration of the initial programme theories based on national stakeholders’ accounts.

#### Data analysis

Interviews were audio-recorded, transcribed verbatim and imported into NVivo 10 software [45]. The framework approach [46] was used to systematically identify contexts (C), mechanisms (M), and outcomes (O) in the interview transcripts and documents, and chart hypothetical relationships between them (C-M-O configurations) to formulate programme theories for each programme component.

The Framework approach is sufficiently open to allow for novel themes to emerge inductively during analysis [47]. First, transcripts were read and re-read (familiarisation), followed by open coding to identify contexts, mechanisms and outcomes. Emergent concepts which did not fit explicitly with the C-M-O framework were also coded. Two researchers (MT and SMH) open-coded three interviews of staff from different parts of the country. The research team then met to compare and contrast codes, clarify understanding of contexts, mechanisms and outcomes, and agree on an initial coding framework. Two independent coders (FR, KON, researchers who had recently joined the research team) were invited to analyse four interviews (from different professional backgrounds and locations) to further refine the coding framework. This coding framework was applied to subsequent transcripts by the research team. Framework development was a dynamic process with regular meetings to discuss new codes or merging existing codes, assumptions, and ideas about C-M-O configurations.

Having openly coded all of the transcripts, data were sorted and synthesised by theme bringing similar concepts together (thematic charting). At this stage, themes were sorted under the individual programme components: national working group (SMH), retinopathy screening (MT), national foot care model (KON) and national model of integrated care (FR). Each researcher led on the synthesis of codes and development of themes for a different programme intervention. This facilitated data management but also enabled data immersion necessary to develop a programme theory about the relationship between contexts, mechanisms and outcomes for that intervention. In some instances, participants themselves outlined partial C-M-O configurations during interviews (e.g. between contexts and outcomes, mechanisms and outcomes); these relationships were refined or revised by examining other participant interviews. However, in most cases, the research team developed C-M-O configurations based on the analysis of all interviews, starting with a synthesis of the proposed outcomes and working backwards to build a theory about the mechanism that led to that outcome and the context that triggered the mechanism.

Open-ended responses to the conference survey were coded using the same approach although emergent themes tended to reiterate, and overlap with enabling and disabling contexts identified during the interviews. The themes were used to reinforce or refine the initial C-M-O configurations.

Memos were used and shared throughout the analysis to note assumptions, events and changes in the NDP, coding definitions, hunches and early impressions [48]. The language and expressions of the participants were maintained as far as possible, using in vivo codes, to avoid losing the meaning and context. The results were presented to the wider research team for discussion.

### Stage 2: data collection to test programme theories

The aim of stage 2, which is currently underway, is to collect data to test the C-M-O configurations developed in stage 1. A multiple case study design is being used. Case studies are often used in realist evaluation [34, 49–51]; this approach emphasises the in-depth study of phenomena in their real-life context, and the importance of theory to inform the design, selection and interpretation of case studies [52].

#### Case selection

**Fig. 3:**
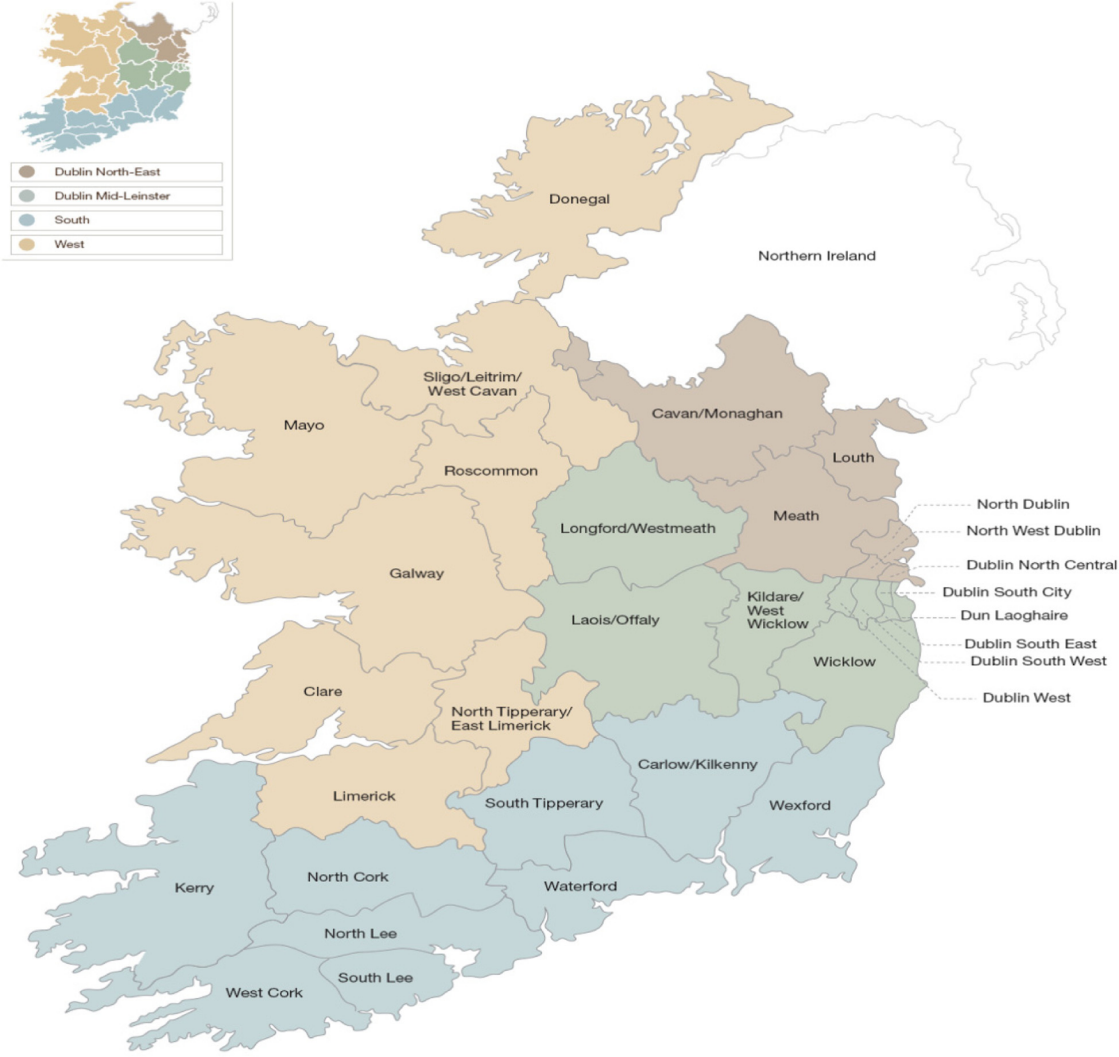
Map of administrative regions in the Health Service Executive (HSE)

A case was defined as a geographical area within one of the four HSE administrative regions (Fig. 3).

A number of criteria were used to select a case area. Firstly, cases had to have received an intervention from the NDP (retinopathy screening programme, integrated care nurse and/or podiatrist). Second, it emerged during stage 1 analysis that the presence or absence of a diabetes initiative (either a primary care-led diabetes initiative, an existing community diabetes nurse specialist (DNS) service, or an established diabetic retinopathy (DR) screening initiative) was an important contextual factor; therefore, we theoretically selected cases on this basis. More detail on the underlying rationale for case selection is available (see Additional file 3).

We mapped these criteria for all areas, starting with the allocation of interventions by the NDP, and discussed the selection of cases within the research team. Table 2 outlines the final selection of four cases and their characteristics.

**Table 2:** Case study selection & sampling criteria

Case 1	Case 2	Case 3	Case 4
NDP interventions • Received 2 components Existing infrastructure and engagement • Prior DR screening initiative delivered in the community, open to patients in a select number of general practices (no universal access) • Existing primary care diabetes initiative with voluntary enrolment by some general practices	NDP interventions • Received 3 components Existing infrastructure and engagement • Previous population-based retinopathy screening initiative offered to all general practices in the area • Existing primary care diabetes initiative with voluntary participation from some practices • Community DNS	NDP interventions • Received 3 components Existing infrastructure and engagement • Previous hospital service which provided eye screening for those referred to service, no population-based screening programme in the community • Existing primary care diabetes initiative with voluntary participation from general practices • Community DNS	NDP interventions • Received 3 components Existing infrastructure and engagement • Previous hospital service which provided eye screening for those referred to service, no population-based screening programme in the community • No existing primary care-led diabetes initiative • Community DNS

#### Data collection

Within each case, multiple sources of data will be used to test the C-M-O configurations developed in stage 1.

##### Qualitative

Qualitative interviews will be undertaken with key stakeholder groups purposively sampled in each case and this work is ongoing. Table 3 outlines the expected number of participants per case. In each area, local members of the regional DSIG will be invited to participate. Participants will be invited to suggest other stakeholders such as health service managers that they engage with. These managers may or may not be formally involved with the DSIG but have a role in deploying and coordinating resources. Integrated care nurses and podiatrists appointed as part of the implementation of the NDP will be invited to participate in an interview or focus group, as well as diabetes nurse specialists (hospital and community-based) and podiatrists previously in post. A purposive sample of GPs and practice nurses will be recruited from practices enrolled/not enrolled in primary care-led diabetes initiatives. Participants will be recruited through educational meetings, continuing professional development groups and local DSIGs. People with diabetes will be recruited using a web-based entry form and dedicated telephone line (participant portal). To ensure we have patient representation specific to our cases, we will ask participating healthcare professionals to publicise our study and the participant portal to patients, and display sign-up posters in local clinics. People with diabetes will also be recruited through local education and awareness events run by a national patient advocacy group (Diabetes Ireland).

**Table 3:** Stage 2 sample per case

	Number per case	Total
DSIG member	2	8
Endocrinologist	1	4
General practitioner (GP)	2	8
Practice nurse	2	8
Diabetes nurse specialist/integrated care nurse	2	8
Podiatrists	2	8
Ophthalmologist	1	4
Patient representative	2	8
Health service manager	1	4
Total	15	60

A theory-driven topic guide has been devised for interviews in stage 2, based on the programme theories developed during stage one. The topic guide has been tailored to the stakeholder group being interviewed (hospital specialist, GP, practice nurse, specialist nurse, podiatrist, person with diabetes). During the interview, we will have an active and explicit role in explaining the contexts and outcomes of interest, to ensure a shared understanding of the terminology and purpose of the questions. In the context of our developed theories, participants will be invited to explain how their experience fits with that theory and reflect on what may explain the outcomes in their area [53]. The topic guide has been piloted with a convenience sample of one GP and two practice nurses, staff who would be most familiar with or in receipt of most programme components. Written consent will be obtained prior to each interview, and all interviews will be audio-recorded and transcribed verbatim. Thematic analysis of the interviews will be guided by the initial programme theories identified in stage 1. However, analysis will be open to emergent themes to facilitate further theory refinement.

##### Quantitative

To assess programme outcomes, administrative data and healthcare professional surveys will be analysed (see Table 1). For the national retinopathy screening programme, the outcomes being examined are registration, consent and uptake. These will be examined using local audits of clinical records in general practice. For the national foot care model, case-specific activity data including the number and risk profile of patients will be analysed. For the national model of integrated care, a national survey of diabetes nurse specialists (including integrated care nurses) is being conducted. This will be supplemented with case-specific analysis of activity data, including the number of GPs engaging with the integrated care nurse service and the number of patient consultations.

### Stage 3: analyse data to interrogate theories

In realist evaluation, the unit of analysis is the theories hypothesising the mechanisms by which an intervention produces certain outcomes in a particular context [54]. A matrix will be used to analyse and synthesise both the qualitative and quantitative data available for each case (administrative data, survey data, transcripts) [55, 56]. A matrix will be constructed for each programme theory relating to various programme components (retinopathy screening, national foot care model, and national model of integrated care). Following the example of O’Cathain and colleagues [57], the columns of the matrix will contain the contexts, mechanisms and outcomes for a given theory. Each row in the matrix will represent a different case (see Additional file 4 for an example). This approach will facilitate within-case analysis, highlighting similarities or discrepancies between data sources which may lead to further data collection or analysis [56]. It will also facilitate cross-case analysis to identify patterns across cases. NVivo 10 software will be used to store and manage data [45].

### Stage 4: interpret analysis to refine or revise the initial programme theories

In light of the analysis in stage 3, the programme theories underpinning the NDP will be refined. The original programme theories will be assessed against the evidence emerging within cases and then between cases to more accurately reflect the experiences of those delivering and receiving the changes introduced by the NDP, and the outcomes realised in different contexts.

#### Ethical considerations

This study has been approved by the Clinical Research Ethics Committee of the Cork Teaching Hospitals. Each participant in the study is asked for written informed consent prior to conducting the interviews and focus groups. Informed consent has also been sought from survey participants, and data have been anonymised for analysis and reporting. Permission has been granted by the NDP to analyse activity data submitted to the programme. Anonymity will be assured at each case study site and all participants will be given a unique ID number. Initial programme theories were presented to members of the national working group for comment. Results from later stages will be fed back to case study participants in the form of a case report. Any potentially identifiable information will be removed prior to reporting and publishing the findings.

## Discussion

Realist evaluation, which allows for the study of context and its influence on outcomes, is appropriate for examining the implementation of the NDP, given the history of regional variation in diabetes services in Ireland. This paper outlines the protocol for a mixed methods evaluation to explore which aspects of the programme are working, for whom and in which circumstances.

Geographic case studies are often difficult to define [52], and this has been a particular challenge in this study given the ill-defined boundaries of health services in Ireland. Catchment areas for health services and hospitals are often fluid, and the organisational structures within the health service have gone through several recent reconfigurations. Furthermore, the results of stage 1 of this study suggest variation in diabetes services within regions and counties depending on the local resources, infrastructure and engagement from stakeholders such as GPs and local management. We have selected cases for stage 2 on the basis of these preliminary findings. The aim of this study is to understand how the NDP is working, for whom and in what circumstances. Therefore, the cases are considered instrumental as opposed to intrinsic [58], that is they are being used to gain a deeper understanding of programme implementation as a whole, as opposed to focusing on the uniqueness of the individual case itself.

We have used a number of strategies to enhance the rigour of this study. Data collection tools including topic guides and surveys have been extensively piloted. Different triangulation techniques will be used to strengthen the validity of findings, including the use of mixed methods, multiple data sources (interviews and documents) and researchers from different disciplinary perspectives (health services research, epidemiology, public health, clinical medicine) [59]. While the limitations of member checking as a strategy to verify overall results have been highlighted, it is considered appropriate to enhance validity in case study research, as case reports maintain the contextual information that allows participants to relate their experiences to synthesised results [52, 60].

Throughout this study, data will be collected and analysed concurrently within each stage to allow emergent lines of enquiry to be explored [60]. The research team has endeavoured to be responsive to the implementation of the programme and changeable context in which it is being rolled out. For example, in October 2015, the Department of Health in Ireland agreed a new contract with GPs which provided financial reimbursement for two structured diabetes review visits in general practice per year. This scheme is known as the Diabetes Cycle of Care. Patients with type 2 diabetes who have a medical card or a GP visit card, which entitles them to free GP care in Ireland, are eligible to be registered by their GP for the scheme. This is a significant influential factor in the context of the NDP. Although not part of the initial programme theories, given its recent introduction, we have adapted our topic guide to explore how the introduction of this financial incentive may influence implementation.

Complex social interventions such as the NDP achieve their outcomes by active input from various stakeholders. Qualitative research is an important part of exploring the reasoning and responses of stakeholders to a programme [37]. Similar to other realist evaluations [61], the results of our interviews with national programme stakeholders, who were also local implementers with context-specific experience, further refined ‘official’ programme theories about which aspects are working, in which circumstances and why. This evaluation builds on previous work by the research team which analysed the many diabetes care policies in Ireland, thereby providing information on some of the contextual factors that preceded the national programme [24]. Pawson suggests that by defining clearly the boundaries of case studies, evaluators are then able to harness the potential of administrative data, for example, relevant to quantifying the outcomes of programmes in realist evaluation [37]. Collaboration with the NDP has enabled us to analyse such administrative information where available. However, we are limited by the lack of a diabetes register in Ireland or national databases on the quality of diabetes care, and patients’ health service interactions and outcomes.

There is increasing interest in the evaluation of health policy and health service implementation. In particular, there is increasing emphasis on theory-based evaluations which aim to establish the context and mechanisms that facilitate successful implementation rather than simply focusing on the achievement of specific endpoints [35]. Realist evaluation has been used for this purpose to study a diverse range of service changes including the introduction of an integrated care pathway for palliative care [41], a multifaceted maternity care programme [49], ‘communities of practice’ [38], oncology teams [54] and quality improvement in primary care [51]. There are very few evaluations of the implementation of programmes or service interventions in Ireland, and to our knowledge, this is the first realist evaluation of a programme in Ireland.

The NDP is constantly moving between planning for future work streams and ongoing implementation of the current work streams. Therefore, the programme offers a potentially unique opportunity to evaluate and inform the implementation of changes in the Irish health system as they emerge and evolve. For example, there has been phased recruitment of integrated care diabetes nurse specialists (known as integrated care nurses (ICNs)) to support the implementation of the national model of integrated care as resources have been secured at national level. A protocol has been developed to clarify the role of the ICN, partly in response to barriers to implementation highlighted in the evaluation. There is close collaboration between the national working group and research team; the principal investigator (PK) is a member of the working group which provides an opportunity to provide formative feedback on implementation to those responsible. Our results should also provide insights relevant to the implementation of other clinical care programmes in Ireland operating in similar contexts. Furthermore, we hope that the findings will be relevant to programmes in other countries, some of which are also evaluating implementation of new care programmes [62].

## Additional files

Additional file 1: Secondary sources of information included in documentary analysis. Description of data: details of documents used in documentary analysis during stage 1 (DOCX 24 kb)

Additional file 2: Topic guide stage 1. Description of data: topic guide used during semi-structured interviews in stage 1. (DOCX 22 kb)

Additional file 3: Case selection. Description of data: rationale for case selection. (DOCX 12 kb)

Additional file 4: Sample matrix. Description of data: an example of the matrix approach that will be used to integrate qualitative and quantitative data. (DOCX 11 kb)
