# Supplementary material for: Evaluating the implementation of a national clinical programme for diabetes to standardise and improve services: a realist evaluation protocol
Source: Implement Sci. 2016 Jul 28;11:107. doi: 10.1186/s13012-016-0464-9 (PMC4964144; doi:10.1186/s13012-016-0464-9)
Supplement: Supplementary file 1 — Secondary sources of information included in documentary analysis. Description of data: details of documents used in documentary analysis during stage 1 (DOCX 24 kb) [file 13012_2016_464_MOESM1_ESM.docx]

Additional file 1:

Table 4 Secondary sources of information included in documentary analysis

| Type | | Source |
| --- | --- | --- |
| **National-level Policy 2010-2015** | | |
| National Service Plans | Health Service Executive 2011 - 2015 | |
| ‘Model of Care for the Diabetic Foot’ | Health Service Executive National Diabetes Programme Clinical Strategy and Programmes Directorate, 2011. | |
| ‘Standards for Quality Assurance in Diabetic Retinopathy Screening’ (1^st^ Ed.) | Diabetic RetinaScreen. The National Diabetic Retinal Screening Programme (DRSP).(n.d.) | |
| ‘Clinical Practice Guidelines for Treatment Clinics’ (1^st^ Ed.) | Diabetic RetinaScreen. The National Diabetic Retinal Screening Programme (DRSP). (n.d.). | |
| ‘Framework for the Development of a Diabetic Retinopathy  Screening Programme for Ireland’ | Health Service Executive, 2008. | |
| ‘A Practical Guide to Integrated Type 2 Diabetes Care’ | Irish College of General Practitioners. Version 5 Final Version. (2013). Unpublished document. | |
| ‘Diabetes Expert Advisory Group. First Report.’ | Health Service Executive, 2008. | |
| **Audit reports** |  | |
| Audit of compliance with the HSE Model of Care for the Diabetic Foot in high risk diabetic patients in selected acute hospitals- Summary report | Health Service Executive Quality Assurance and Verification Division, 2015. | |
| Diabetes in General Practice. Audit Report. March 2009 - June 2010. | Murphy K., McHugh S., and Moran J. Diabetes in General Practice, 2010. | |
| East Coast Area Diabetes Shared Care Programme (ECAD). Audit 2006. | Health Service Executive Mid-Leinster, 2008. | |
| Audit Report of the HSE Midland Diabetes Structured Care Programme | Marsden P., Brennan C., McHugh S., and V. Harkins. Health Service Executive, 2010. | |
| **HSE National Clinical Programme Documentation 2010 - 2015** |  | |
| Role of the Clinical Nurse Specialist in Diabetes-Integrated Care (Draft) | Health Service Executive National Diabetes Programme Working Group (n.d.). Unpublished document. | |
| Presentation. Dr Barry White, National Director of Clinical Strategy and Programmes. | **White, B. (2012).** National Clinical Programmes Clinical Programmes and Strategy. Presentation at the **M.Sc. in Health Services Management** Annual Guest Lectures 2012 **Wednesday 30th May to Friday 1st June**. The Impact of Health Reforms – Opportunities and Challenges, Trinity College Dublin, Ireland. | |
| Background Information on the National Clinical Programmes: Mission, Vision and Objectives (Presentation) | Health Service Executive National Diabetes Programmes. [Online]. Available from: <http://hse.ie/eng/about/Who/clinical/natclinprog/missionvision.pdf> | |
| Nurse Education Programme | Health Service Executive National Diabetes Programmes. [Online]. Available from: <http://www.hse.ie/eng/about/Who/clinical/natclinprog/diabetesprogramme/streams/nurseed/> | |
| **Media 2010 - 2015** | | |
| Coverage in national newspapers, online health forums | Irish Times. (2013). College of GPs withdraws from HSE clinical programmes in protest over fees. [Online]. Available from:  <http://www.irishtimes.com/news/ireland/irish-news/college-of-gps-withdraws-from-hse-clinical-programmes-in-protest-over-fees-1.1466517>    Irish Times. (2014). We know the best treatment for diabetes. We need funds to deliver it. [Online]. Available from:  <http://www.irishtimes.com/life-and-style/health-family/we-know-the-best-treatment-for-diabetes-we-need-funds-to-deliver-it-1.1956499>  Irish Health. (2012). Diabetes care programme to begin. [Online]. Available from:  <http://www.irishhealth.com/article.html?id=21327>  Irish Medical Times. (2013). New GP contract under review. [Online]. Available from:  <http://www.imt.ie/features-opinion/2013/08/new-gp-contract-is-under-review.html>  Irish Health. (2013). Diabetes care in crisis. [Online]. Available from: <http://www.irishhealth.com/article.html?id=22109>  Irish Medical Times. (2012). Total coverage for diabetes by 2014. [Online]. Available from:  <http://www.imt.ie/news/latest-news/2012/04/total-coverage-for-diabetes-by-2014.html>  Irish Medical Times. (2012). Integrated diabetes care is priority – HSE. [Online]. Available from:  <http://www.imt.ie/news/latest-news/2012/03/integrated-diabetes-care-is-priority-hse.html>  Irish Health. (2014). Diabetes eye screen scheme on target. [Online]. Available from: <http://www.irishhealth.com/article.html?id=23984>  Irish Times. (2014). One-in-8 referrals after eye tests for diabetes. [Online]. Available from:  http://www.irishexaminer.com/ireland/one-in-8-referrals-after-eye-tests-for-diabetes-283273.html  Irish Medical Times. (2014). RetinaScreen targets 145,000. [Online]. Available from:  <http://www.imt.ie/news/uncategorized/2014/08/retinascreen-targets-145000.html> | |
|  |  |  |
| DRSP Presentations | Keegan, D. (2014). Transition to a National Diabetic Retinopathy Screening and Treatment Programme. Presentation at the Royal Victoria Eye and Ear Hospital Research Foundation. New Frontiers in Ophthalmology: Diabetic Retinopathy, Dublin, Ireland.  Acheson, R. (2014). Diabetic Retina Screen - Arbitration and Gatekeeping. Presentation at the Royal Victoria Eye and Ear Hospital Research Foundation. New Frontiers in Ophthalmology: Diabetic Retinopathy, Dublin, Ireland. | |
| **Stakeholder position statements/announcements** |  | |
| DRSP announcement: ‘Diabetic RetinaScreen has begun new information and advertising campaign.’ | Diabetic RetinaScreen. The National Diabetic Retinal Screening Programme (DRSP). (2014). [Online]. Available from: <http://www.diabeticretinascreen.ie/news-events/diabetic-retinascreen-has-begun-new-information-and-advertising-campaign.418.html>. | |
| HSE announcement: ‘17 new Integrated Care Diabetes Nurse Specialists to support patients with diabetes.’ | Health Service Executive. (2013). [Online]. Available from: <http://www.hse.ie/eng/services/news/media/pressrel/newsarchive/2013archive/Mar2013/diabetesnursespecialist.html> | |
| Statement to ICGP members on the HSE Clinical Care Programme for Integrated Diabetes Care. | Irish College of General Practitioners, Clinical Care Programmes Committee. (2013). [Online]. Available from: <http://www.icgp.ie/go/about/policies_statements/2013/720A1ABB-9266-83F9-D39D851FD9EDD2F3.html> | |
| HSE summary ‘National Clinical Programme for diabetes’ | Health Service Executive. (n.d.). [Online]. Available from: <http://www.hse.ie/eng/about/Who/clinical/natclinprog/diabetesprogramme/diabetesprogramme.html> | |
| RCPI summary ‘About the National Clinical Programmes’ | Royal College of Physicians of Ireland. (n.d.). [Online]. Available from: <https://www.rcpi.ie/article.php?locID=1.10.410.496> | |
| ICGP announcement: ‘ICGP highlights to Minister for Health need for focus on chronic disease management in general practice.’ | Irish College of General Practitioners. (2015). [Online]. Available from: <http://www.icgp.ie/go/about/policies_statements/2015/1FBD6E2A-E3A6-11BA-8253A1084DC2EA93.html> | |
| IDNSA announcement: ‘National Integrated Care Programme’ | Irish Diabetes Nurse Specialist Association. (2014). [Online]. Available from: <http://www.idnsa.ie/news/national-integrated-care-programme/> | |
| **DRSP official documentation** |  | |
| Letter to Health Care Professionals, Patient Consent Form, Information Leaflet, Screening Charter, Treatment Clinic Locations Listing | [Online]. Available from: <http://www.diabeticretinascreen.ie> | |
| **Other 2004-2015** | | |
| Parliamentary Questions relating to diabetes | [Online] Available from: <http://www.oireachtas.ie/parliament/oireachtasbusiness/parliamentaryquestions/>  [Online] Available from: <http://oireachtasdebates.oireachtas.ie/?readform> | |
| Oireachtas debates relating to diabetes |  |  |
