# Supplementary material for: Evaluating the implementation of a national clinical programme for diabetes to standardise and improve services: a realist evaluation protocol
Source: Implement Sci. 2016 Jul 28;11:107. doi: 10.1186/s13012-016-0464-9 (PMC4964144; doi:10.1186/s13012-016-0464-9)
Supplement: Supplementary file 2 — Topic guide stage 1. Description of data: topic guide used during semi-structured interviews in stage 1. (DOCX 22 kb) [file 13012_2016_464_MOESM2_ESM.docx]

**Evaluation of the Implementation of the National Diabetes Programme**

**Topic Guide Stage 1**

| **National Clinical Programme for Diabetes** |
| --- |
| **Rationale:**  The purpose of this research is to document and evaluate the change process surrounding the implementation of the NDP in the Irish health system. We would like to understand how the programme is working and what is involved in the implementation of the different work streams. |

The interview should last between 30-45 minutes.

Just some general house-keeping before we start (Briefly go through consent form)

- If it is ok with you I will audio record the interview so I can give you my full attention and don’t have to take any notes. This way I can be sure I don’t miss anything.
- Anything we discuss will be confidential and your identity will remain anonymous on any reports or publications. We may use direct quotes from this interview but again I stress that your name will **not** appear anywhere. Your identity and position will be kept completely anonymous.
- Finally you can stop the interview at any point, if you wish. And you are free to withdraw from the study at any time.
- During the interview, I will refer to the NDP as the ‘programme’.
- Do you have any questions for me before we get started?
- Sign consent and give copy.

| **TOPIC GUIDE** | | | |
| --- | --- | --- | --- |
| **BACKGROUND QUESTIONS** | **PROBES/PROMPT** | | |
| Can you tell me a little about your role and how you are connected to the programme? | - How long in role? - How did you become involved? Why did you take on role? - Has role changed? If so, how? - Who do you report to? | | |
| **ACCOUNTS OF THE PURPOSE AND KEY ASPECTS OF THE NCPD** | | | |
| Why was the programme set up? | | - What drove the need for change?   - Events that led to/triggered the programme   - Problem areas? - Possible drivers: Individual/ health system/ economic; related complications, increasing prevalence, ageing population, EAG report, Regional variations | |
| **HOW IT IS EXPECTED TO WORK** | | | |
| Can you tell me the purpose of the programme?  OR the individual components  DNS (Integrated Care Programme)  Retinopathy Screening Programme  Model of care for the diabetic foot  What are the national diabetes programme responsible for? | | - Does it have specific aims? - Prompt-   - Access to diabetes services (screening, podiatry, GP, consultants)   - Quality of diabetes care (staffing, skills, patient safety& satisfaction)   - Overall cost (reduce healthcare cost/ cost effectiveness) | |
| Can you describe the key components of the programme?  How do these changes fit into existing services? | | How do they work in reality? | |
| **IMPLEMENTATION OF THE PROGRAMME** | | | |
| What stage of the implementation process is the programme currently at?  AND the individual components (if not mentioned)  DNS (Integrated Care Programme)  Retinopathy Screening Programme  Model of care for the diabetic foot  *Probe national stakeholders to talk about each work stream*  What are the next steps? | | - Prompts: Nationally, regionally, locally - Plan/strategy in place for implementation   - *(who decided, who involved)* - Implementation progressed/progressing?   - *Areas that are better, roadblocks, any changes evident?*   How is implementation co-ordinated   - - *(Communication, organisation, flow on info, adapted plans)*   How is progress monitored- (KPI’s)- what are they?   - Who’s responsible for monitoring implementation? | |
| How were the work streams prioritised?  How is/was funding allocated? | | Prompts: Resources, EAG recommendations   - Decision making process (who was involved) - How do the programme decide how to spend money? | |
| How do the regional DSIGs fit with national programme? | |  | |
| **NCPD FACILITATORS AND BARRIERS** | | | |
| Can you tell me what has gone well/is going well/less well in the implementation of the programme overall?  AND  DNS (Integrated Care Programme)  Retinopathy Screening Programme  Model of care for the diabetic foot  In your opinion what things will enable the changes to happen? | | - What elements are working? - What elements are not working? - Why is this? - Probes: Facilitators/barriers   Staffing  Finance  Resources  Patient satisfaction |  |
| Have there been any changes to the implementation process that were not foreseen? Tell me about that… | | - Why? *(resources, cost, time)* - Adapting the implementation *(Why?)* - Any issues/concerns about changes *(resolved, impact implementation, delay)* |  |
| **ANTICIPATED OUTCOMES** | | | |
| What will be the impact of the NCPD be?  What do you think will change? | | - Positive/Negative impact - Health System Outcomes: *(Shift diabetes care from secondary care to primary care, reducing over cost, reducing resource use, improving access and quality of care)* - Service provider Outcomes: *(GP, DNS, podiatrist, etc. standardised care, care managed the same across the country)* - Patient outcomes: *(high quality care, access to services, improvement in diabetes)* | |

| **TO FINISH** |
| --- |
| Is there anything that I haven’t touched on that you think is important?  Are there any other issues you would like to discuss? |
| Are you aware of any relevant material /documents that could be of use or other people who might be useful to contact? |
| If I need to clarify anything from today can I call you and maybe in the future when implementation progresses and things change I might come back to you to get your thoughts on that. |
