# Supplementary material for: Evaluating the implementation of a national clinical programme for diabetes to standardise and improve services: a realist evaluation protocol
Source: Implement Sci. 2016 Jul 28;11:107. doi: 10.1186/s13012-016-0464-9 (PMC4964144; doi:10.1186/s13012-016-0464-9)
Supplement: Supplementary file 3 — Case selection. Description of data: rationale for case selection. (DOCX 12 kb) [file 13012_2016_464_MOESM3_ESM.docx]

Additional File 3

Case selection

There are four administrative regions in the Health Service Executive (South, West, Dublin-Mid Leinster, Dublin-North East). A Diabetes Services Implementation Group (DSIG) was established in each region. However, given the geographical spread of each administrative region, it was necessary to define a case as a smaller area (county/county groupings) within a region (n = four areas, one per region). Furthermore, results from stage 1 suggested that administrative regions were not homogenous; areas within a region varied substantially in terms of diabetes services, resources and infrastructure in place prior to the National Diabetes Programme. And, while all regions are in receipt of the national retinopathy screening programme, additional programme resources such an integrated care nurse (ICN) or a podiatrist were not allocated to entire regions but rather to smaller areas such as counties or groups of counties (e.g. Cork/Kerry).

In addition to an *a priori* selection criterion (areas which had received an intervention from the national diabetes programme [retinopathy screening programme, integrated care nurse, and/or podiatrist]), we selected cases on the basis of another criterion which emerged consistently as an important contextual factor during stage 1 interviews: the presence of a pre-existing diabetes initiative, either a primary care-led diabetes initiative, a community diabetes nurse specialist (DNS), or a diabetic retinopathy (DR) screening initiative.
