# Supplementary material for: Evaluating the implementation of a national clinical programme for diabetes to standardise and improve services: a realist evaluation protocol
Source: Implement Sci. 2016 Jul 28;11:107. doi: 10.1186/s13012-016-0464-9 (PMC4964144; doi:10.1186/s13012-016-0464-9)
Supplement: Supplementary file 4 — Sample matrix. Description of data: an example of the matrix approach that will be used to integrate qualitative and quantitative data. (DOCX 11 kb) [file 13012_2016_464_MOESM4_ESM.docx]

Supplementary file

Table XX: Dummy Matrix

Programme Theory 1:

| Case | Context 1 | + Mechanism 1= | Outcome of Interest:  E.g. adoption of integrated care nurse service |
| --- | --- | --- | --- |
| 1 |  |  |  |
| 2 |  |  |  |
| 3 |  |  |  |
| 4 |  |  |  |
